# End-of-life care research with bereaved informal caregivers – analysis of recruitment strategy and participation rate from a multi-centre validation study

**DOI:** 10.1186/s12904-015-0020-4

**Published:** 2015-05-02

**Authors:** Stephanie Stiel, Maria Heckel, Sonja Bussmann, Martin Weber, Christoph Ostgathe

**Affiliations:** Department of Palliative Medicine, Universitätsklinikum Erlangen, Friedrich-Alexander-Universität Erlangen, Krankenhausstraße 12, 91054 Erlangen, Germany; Comprehensive Cancer Centre, CCC Erlangen - EMN, Universitätsklinikum Erlangen, Friedrich-Alexander-Universität Erlangen-Nürnberg, Erlangen, Germany; Interdisciplinary Palliative Care Unit, III. Department of Medicine, University Medical Centre of the Johannes Gutenberg University of Mainz, Mainz, Germany

**Keywords:** Quality of dying and death, Validation, Assessment tool, Palliative care, Recruitment, Research participation, Informal caregiver, Participation rate, Willingness, Motivation, Distress

## Abstract

**Background:**

One essential element of research is the successful recruitment of participants. However, concerns are obvious regarding the ethical implications of involving terminally ill and even dying patients and their informal caregivers as research participants. This study aims to illustrate central issues encountered when recruiting bereaved informal caregivers for a questionnaire validation study on the quality of dying and death.

**Methods:**

Between July 2012 and November 2013, informal caregivers of deceased inpatients who were treated at two palliative care units in Germany were invited to participate in a questionnaire validation study. Informal caregivers were called by a trained researcher at the end of the fourth week after death at the earliest and by the sixteenth week after death at the latest and asked to participate in a face-to-face interview in their private home.

**Results:**

The overall participation rate of all eligible informal caregivers was 76.1% (226/297). The mean burden score was 2.5 (NRS from 0 = no burden to 10 = maximum burden; n = 221). Higher burden scores (≥4) were associated with emotional and burdensome memories (n = 34) being invoked throughout the interview. Severe or maximum burden scores (≥7) were stated by 13.2% of participants. The average time between the associated patient’s death and the informal caregiver’s interview was 57.3 days (range 26–176 days, median 49.5 days). 5.3% of all 226 interviews were not completed due to different reasons. Participants’ comments on the way in which the study was conducted gave insight into their motivation to take part in the study and their evaluation of the interview situation.

**Conclusions:**

The recruitment strategy can be recommended to other researchers developing research with bereaved carers. The burden caused by study participation was acceptable to the researchers carrying out this research, although a small group of participants experienced high levels of burden which should be anticipated and appropriate support services offered. Family caregivers are willing to support end-of-life care research, have different motivations for participation and even reported benefits from participation. Nevertheless, study designs have to take into account and ease the potential burden of interviews for caregivers experiencing grief.

## Background

End-of-life care research is important in order to improve the quality of treatment, quality of care and quality of life for patients in a palliative care situation and for their loved ones [1]. Research may also foster care providers’ understanding of individuals at the end of their lives. To ensure that further developments and improvements can be made in end-of-life care, substantial research is necessary. Similarly to other specialist areas of medicine, end-of-life care practice should be based on evidence. Rees and colleagues describe research in end-of-life care as a “minefield of ethical issues” due to the particular vulnerability of the population [2].

Indeed, end-of-life care research embodies several potential challenges, barriers and general issues. One essential element of clinical research is the successful recruitment of participants who comply with all study inclusion and exclusion criteria [3]. However, concerns are obvious regarding the ethical implications of involving terminally ill and even dying patients and their informal caregivers as research participants [4]. These vulnerable study populations are considered particularly at risk of being burdened by research participation [5]. Although we strive for high sample sizes and response rates in general, the desired sample size in end-of-life care research should be balanced between the actual need to recruit many participants to ensure reliable results, on the one hand, and the ethical weighing up of risks versus benefits for each participants and of expenses of the research team and participants versus potential outcomes for the science, on the other hand, especially in projects of a very sensitive nature.

Furthermore, conducting research with informal caregivers of individuals receiving palliative care is challenging and requires careful ethical and methodological decision-making. With regard to specific recruitment characteristics for informal caregivers, some researchers appear to be able to recruit participants quickly and without significant issues, while other researchers have more difficulty attaining the planned number of targeted participants [6]. Nevertheless, there is a need to recruit bereaved caregivers, for example to obtain information about the quality of care in the dying phase in order to improve the quality of patient care.

In spite of guidelines pertaining to ethical concerns in end-of-life research [7], no specific recommendations exist as to how researchers may overcome these challenges [8]. A first systematic review concerning the perspectives of both patients receiving end-of-life care and of their families with regard to research was published in 2010. This publication highlighted important factors to be considered when selecting research participants and the types of research design that would generally lead to approval/support or rejection. The authors White and Hardy conclude that patients and family caregivers have a high willingness with regard to, and may actually benefit from, participation in end-of-life care research [9]. The current published literature provides some individual reports of successful efforts to recruit patients and family caregivers in a palliative care situation which may serve as helpful examples when designing end-of-life care research. Overall, the distress caused by study participation was reported to be rather low in several studies [10,11].

### Study aim

This article aims to enlarge the current knowledge base and to illustrate some issues encountered when recruiting bereaved informal caregivers as participants for a questionnaire validation study on the quality of dying and death of a deceased close relative or friend [12]. The recruitment method, which considers inclusion and exclusion criteria for potential participants and a detailed description of the actual respondents will be presented. This article discusses the practical and ethical issues surrounding informal caregivers’ participation in quantitative end-of-life care research.

The secondary aim of this paper is to describe an effective recruitment strategy used in end-of-life care in order to determine appropriate forms of end-of-life care research and to assist other researchers and clinicians in developing realistic studies that are feasible and can be successfully implemented with this population.

## Methods

### Study design and recruitment strategy

When defining the recruitment strategy, several aspects had to be decided upon. The most important are shown in Table 1 and will be reflected upon in the discussion section.

**Table 1:** **Relevant aspects of decisions for recruitment strategy in quantitative end-of-life care research**

**Aspect**	**Example of our study**	**Decision**
Implications of study design	Validation	Multi- centre (different approach necessary?)
		High number of participants required
Exact definition of participants	Informal caregivers	Persons who are close to patients and who have cared for the deceased during his/her last days of life on the palliative care unit in any kind of organisational, physical or emotional manner. They might be family members, spouses, companions, friends or neighbours
Requirements for study participants	Complexity of questionnaire/questions	Language skills
	Degree of abstraction	Cognitive capability
	Amount of items/ questionnaires	>18 years old
	Sophistication of instrument	Feel emotionally stable
	Estimated duration of interview	
	Themes	
Time of invitation	During bereavement phase vs. immediately upon contact with palliative care unit	At the end of the fourth week after death at the earliest and by the sixteenth week after death at the latest based on ethical considerations
Time of interview	Bereavement vs. memory accuracy	Four weeks after death at the earliest
Place of interview	Ease of remembering vs. re-experiencing	Participant’s decision of where to meet

Between a) July 2012 and August 2013 and between b) July 2012 and November 2013, informal caregivers of deceased inpatients who were treated at two palliative care units in a) Erlangen and b) Mainz, Germany, were invited to participate in a questionnaire validation study. Here, informal caregivers were defined as persons (family members, close friends or significant others) who were close to patients and who had cared for the deceased during their last days of life in the palliative care unit in any kind of official or legal, physical or emotional manner. They might be family members, spouses, companions, friends or neighbours.

For all deceased patients in the two palliative care units, inclusion and exclusion criteria (Table 2) were checked by two researchers who had access to clinical care information for research purposes as both research units were part of the palliative care departments. Where informal caregivers existed and contact information was known, they were called by a psychologist and trained researcher (Erlangen: SS/MH; Mainz: SB/SM) at the end of the fourth week after death at the earliest and by the sixteenth week after death at the latest. This time was chosen taking all arguments of balancing a potential time of mourning versus possible accurate retrospection due to receding memory into consideration [13,14]. Again inclusion and exclusion criteria were checked. Reasons for refusal were documented.

**Table 2:** **Inclusion and exclusion/drop-out criteria**

**Inclusion criteria**	**Exclusion and drop-out criteria during application of study**
• Patient was at least 18 years old.	• Informal caregiver experiences emotional distress or lack of resilience.
• Patient died no earlier than on the third day of inpatient stay (including day of admission and day of death) so that caregivers had the opportunity to visit the patient and experience quality of dying and death in the palliative care environment.	• Informal caregiver is not sufficiently able to understand and speak German.
• Informal caregiver is at least 18 years old.	• Informal caregiver is cognitively impaired.
• Informal caregiver is sufficiently capable of speaking and understanding German.	• Informal caregiver has withdrawn his/her informed consent.
• Informal caregiver has had personal contact with the patient during the last seven days of life in the palliative care unit.	• Informal caregiver cannot be contacted.
• Informal caregiver has signed informed written consent for study participation.	

This first phone call was intended to inform potential participants in detail about the project design, its scope and aims, potential risks of participation, relevant data protection provisions and the principles of voluntary participation. Carers were given time to consider participation and ask further questions and they were given the option to think about it and call back by themselves or to be called again at a time of their choosing. If desired, the written study information and consent form were posted before the personal meeting during which the interview was carried out. Most carers gave verbal consent during the phone call and written consent before participating in the interview at the face-to-face meeting.

In the event that informal caregivers of deceased inpatients felt unsure of whether they should make an appointment to participate in the study during the first phone call, they were asked whether the researchers were allowed to call again at later point in time of their choosing to ask about their willingness to participate in the project. This gave informal caregivers time to think about it and a second opportunity to raise questions about the study. If they agreed to take part in the second phone call an appointment was made; if they refused during the second phone call they were not called again.

If they provided consent, participants were invited to a face-to-face interview with a trained researcher (Erlangen MH, SS; Mainz SB, SM) either at the palliative care unit or in their private home. None of the caregivers knew the researchers beforehand. The questionnaires were filled in via an interview. In a few cases the caregivers wished to participate immediately on the phone and did not have an extra meeting for this purpose. The researcher carrying out the interview observed the carers’ emotional situation and decided before, during and after the interviews whether any exclusion or drop-out criteria were met based on their clinical experience and depending on carers’ comments.

**Figure 1:**
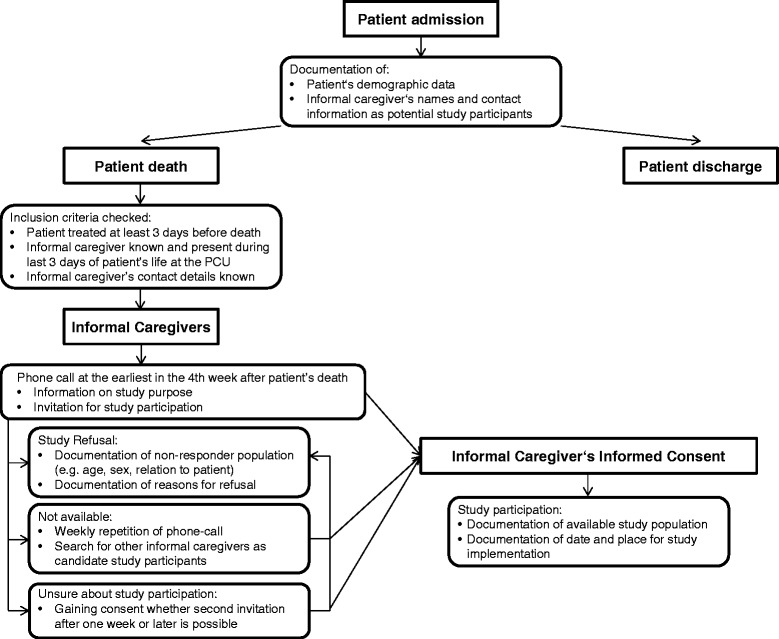
Study design and recruitment strategy.

The study design and recruitment strategy, including criteria for patients and informal caregivers, are shown in Figure 1 in detail.

### Measures

The interviews contained three questionnaires. The Quality of Dying and Death questionnaire, the version for informal caregivers (QoDD) [15] which was to be validated, the Palliative Care Outcome Scale (POS) [16] for external validity testing, and the Patient Health Questionnaire, German version (PHQ) [17], for further testing of the impact of depression and anxiety on the estimation of the quality of dying and death. Details of the validation study will be published elsewhere.

To investigate the balance between the burden and benefit of study participation caused by the use of the QoDD in terms of its validation criteria, all participants were asked to estimate their individual burden on a commonly used but non-validated numerical rating scale from 0 = no burden to 10 = maximum burden [18] (original version: “Overall, how much of a burden did this questionnaire mean to you?”) after completion of the QoDD questionnaire, the last element of the interview. The authors considered burden scores as follows: zero was considered no burden, ten was considered maximum burden as indicated on the scale, one to three were considered mild, four to six moderate, and seven to nine severe burden. Burden scores higher than four (moderate and higher burden) are further investigated here. The time required for each interview (excluding time for confirming consent) was registered by the interviewer.

The informal caregivers’ interviews were supplemented by the evaluation and documentation of personal demographic data such as sex, age, relationship to deceased patient, religion, educational level, and immigrant background.

At the end of the interview, participants were asked to describe what, from their point of view, could have made interview participation methodologically easier (original version: “Is there any way that we could have made this questionnaire easier on you? Probes: Would it have been better to wait longer before we completed this with you? Would it have been better to do it earlier? Should we have asked the questions in a different way?”).

### Data analysis

The software package SPSS 21 (SPSS Inc., Chicago, IL, USA) for Windows was used for statistical analysis of time requirements, burden and demographic data to calculate frequency and descriptive analysis using mean and median values, standard deviation (SD), and range (minimum – maximum).

Participants’ comments on the questions “What could have made participation and study application easier?” and “Do you have any other comments on your study participation?” were noted by the researcher. In cases where study participants indicated a burden higher than four, which corresponds to a relevant moderate burden, they were requested in addition to explain in detail what was especially burdening during study participation (“Can you describe in more detail what it was that you felt was burdening?”) and whether they also experienced benefits from study participation (“Do you think that the interview was in some way helpful to you?”). These questions were included in a written study protocol and asked by both researchers in exactly the same way. The free answers were transcribed word for word by the researchers to ensure they could be documented accurately during quality assessment of the interviews.

All these free text statements are brought together and analysed qualitatively here and have been inductively coded into content categories by two independent researchers (MH and SS). As no disagreement in category development and coding allocation occurred, it was not necessary for a compromise between both coders to be found by a third researcher.

### Ethics

All ethical issues were addressed and discussed within the working group. This study was conducted with the approval of the local ethics committees at both institutions (Erlangen: Ethik-Kommission der Medizinischen Fakultät: 4427 dated 04/13/2011; Mainz: Landesärztekammer Rheinland/Pfalz: 837.379.1 1 (7921) dated 10/24/2011). All participants were informed that they could withdraw their consent at any time during the interview.

## Results

### Recruitment of study participants

Between July 2012 and August 2013, a total of 319 inpatients were treated at the palliative care unit (PCU) in Erlangen, of whom 200 (62.7%) died. In Mainz, 278 inpatients were treated at the PCU during the recruitment period from July 2012 until November 2013, of whom 169 (60.8%) died.

Under consideration of the defined screening criteria (see Table 2), 61 cases of 369 deceased patients were excluded because the patients stayed at the PCU for less than 3 days; 7 were excluded because no informal caregivers were known and a further 11 cases in which no cognitively capable or adult caregivers were available were also excluded.

The remaining 157 (Erlangen) and 140 (Mainz) eligible informal caregivers (n = 297) were contacted by phone. 48 (16.2%) informal caregivers refused to participate due to their reported emotional burden. The main reasons were that they did not wish to reflect on their memories of the death, or that the invitation was too soon after the death. Others (n = 23; 7.7%) dropped out due to a lack of ability to contact them or other organisational barriers, such as that the caregiver had moved too far away to come in again.

**Figure 2:**
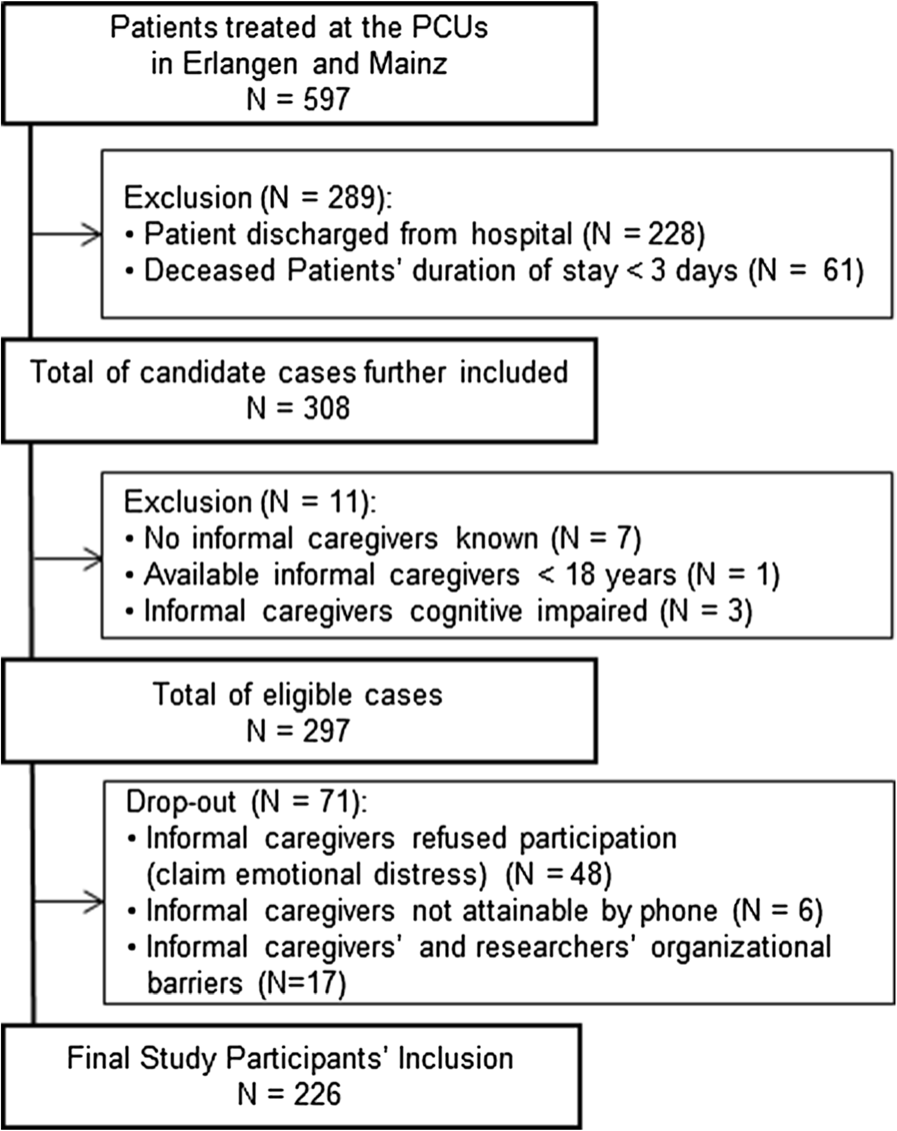
Participant inclusion and exclusion, drop-outs.

As a result 226 study participants (Erlangen: n = 139; Mainz: n = 87) out of 297 eligible cases were able to be included in the study. The overall participation rate of all eligible informal caregivers was 76.1% (226/297) (see Figure 2). Overall, 213 participants (71.7%) consented immediately during the first phone call, but in 21 (9.9%) of these cases the interview did not take place. The reasons were, for example, caregivers’ own illness, caregivers did not send the questionnaire back, or caregivers did not come to their appointment. A further 53 participants were unsure during the first phone call of whether to participate or not. After these candidates were called again, an interview took place in 34 cases (64.2%) and did not take place in 19 (35.8%). None of the final participants withdrew consent during the the study.

### Study sample and implementation

The majority of study participants were female (61.1%), middle-aged (mean 55.6 years old) and of Christian faith (70.4%). Only a small minority had an immigrant background (8.4%). Elementary (28.3%), secondary (27.9%) and tertiary (21.7%) educational levels were almost equally distributed. Most participants were spouses (45.1%) or children (40.7%) of the associated deceased patients (see Table 3).

**Table 3:** **Demographic data of informal caregivers (n = 226)**

**Personal data**	Gender	Female n = 139 (61.1%)
	Age	Mean 55.6 ± 12.8 years
		Range 22.3 to 85.0 years
**Immigrant Background**	Immigrant background	n = 19 (8.4%)
	German nationality	n = 217 (96.0%)
**Religion**	Protestant	39.4%
	Catholic	31.0%
	Muslim	0.9%
	None	22.6%
	Other	1.3%
	Not known	4.8%
**Educational Level**	No education	0.9%
	Elementary school	28.3%
	Secondary education qualification	27.9%
	Qualification for admission to college	14.2%
	Tertiary education qualification	21.7%
	Not known	10.0%
**Relation to deceased patient**	Spouse	45.1%
	Child	40.7%
	Sibling	4.0%
	Friend	1.8%
	Parent	2.7%
	Other relative	4.4%
	Other	1.3%

Most interviews took place by way of a face-to-face meeting (69.0%), 27.0% took place via phone call and in 4.0% of cases the participants wished to fill in the full set of questionnaires themselves and therefore received them by post. The average time between the associated patient’s death and the informal caregiver’s interview was 57.3 days (median 49.5 days, range 26–176 days, interquartile range 31 days). Four (1.8%) interviewees wished to conduct the interview immediately when they received the first contact phone call.

### Burden of and time spent on study participation and perception of study participation

The mean duration of the QoDD application was 39.1 minutes (median 34.1 minutes range 10 – 165 minutes, interquartile range 21 minutes n = 195). For 31 cases only the duration of the whole interview including time spent confirming consent was reported, meaning that these times would lead to overestimation of the duration and are therefore not considered here. 5.3% of all 226 interviews were not completed for reasons of respondent illness (n = 1), poor mental state (n = 4), uncooperativeness (n = 1), the respondent feeling insulted or upset by the questions (n = 1) or unsatisfactory interview conditions such as interview interruptions due to telephone calls to caregivers (n = 5) or the participant’s lack of time or answer via self-assessment.

The mean burden score was 2.5 (SD ± 2.9, empirical range 0–10) for the 221 participants who answered this question. More than half (68.3%) reported no to mild burden ranging from zero to three, 18.6% reported moderate burden, and 24 participants (10.9%) indicated severe burden ranging from seven to nine on the numerical rating scale. Five participants (2.3%) reported maximum burden (10).

With regard to informal caregivers’ ideas and perceptions of their study participation, 216 caregivers provided free text comments on one or more of the open questions. Of these, 252 relevant text fragments answering the actual research question were analysed (see Table 4). We did not analyse free text comments (n = 112) giving general feedback such as “everything was okay” or statements on the quality of general health care. Considering the aspects of how the study was conducted, the time frame was considered to be appropriate (n = 13), although some considered the point in time of participation to be too early (n = 5) or too late (n = 2). Participants who considered the time of the interview appropriate took part between 34 and 164 days (mean 67.9 days, median 49 days) after the patient’s death. Participants who would have preferred the interview to be carried out at a later point in time took part between 34 and 69 days (mean 46.4 days, median 41 days) after the patient’s death and the two participants who wished the interview would have been earlier took part 49 and 59 days after the patient’s death. Coming back to the PCU to participate in the study was perceived as rather emotionally difficult (n = 14) and some informal caregivers evaluated the opportunity to participate by post (n = 1), telephone (n = 1), as a pair of informal caregivers (n = 1) or in a private home (n = 5) as valuable. Challenges and methodological barriers were reported particularly with regard to a lack of insight into the patient’s experience to answer the items (n = 17), inappropriate contents of items such as regarding applied dialysis for prolongation of life in terms of the palliative situation of patients (n = 10) or the high number of items (n = 1). Informal caregivers felt willingness and motivation to participate due to the opportunity to give feedback and contribute to quality management (n = 6), to help the department and give something back (n = 13) or to help other patients and informal caregivers (n = 3). Additionally, many different benefits from study participation, such as the opportunity to talk about and reflect on memories (n = 38), to sort and structure memories and thoughts (n = 8) and to bring the tragedy and bereavement to a conclusion (n = 7), were highlighted by the study participants (see Table 4).

**Table 4:** **Categorisation of 252 codings; multiple answers by individual study participants possible**

**Aspects of conducting the study**		
**Point in time**	• Point in time well chosen	n = 13
	• Point in time rather too early, easier if later	n = 5
	• Point in time rather too late, easier if earlier	n = 2
**Place and atmosphere**	• Difficult to come back to PCU	n = 14
	• Meeting in private home helpful	n = 5
	• Conduction of study by phone easier	n = 1
	• Conduction of study by post preferred	n = 1
	• Attendance of someone close was helpful	n = 1
	• Positive atmosphere during interview	n = 1
	• Amount of time too high	n = 1
**Preferences and improvements**	• All fine	n = 2
	• No additional questions needed	n = 9
	• Other questions preferable	n = 25
**Methodological challenges and barriers**		
**Items**	• Content of items not appropriate	n = 10
	• Lack of insight into patient’s experience to answer items	n = 17
	• Too many items	n = 1
**Answering scales**	• Answering format not appropriate	n = 5
**Other**	• Mixed settings in relevant time impede answering	n = 1
	• Questionnaires not feasible, applicable	n = 5
**Motivation for and benefit from study participation**		
**Motivation**	• To give feedback, contribute to quality management	n = 6
	• To help department, thankfulness	n = 13
	• To help other patients and informal caregivers	n = 3
**Benefit**	• Opportunity to talk about experience and reflect	n = 38
	• Opportunity to sort and structure memories	n = 8
	• Opportunity to come to terms with tragedy	n = 7
	• Increased feeling of security	n = 1
	• Feeling of ongoing care, respecting caregivers’ opinions	n = 4
	• To remember and to awaken memories	n = 4
	• Helpful in general	n = 10
**Burden from study participation**		
**Burden**	• Memories emotional and burdensome	n = 34
	• Not burdensome	n = 3
	• Too many evaluations, wish to be left in peace	n = 1
	• Physical symptoms occurred	n = 1

Results reporting the actual findings from the QoDD and its psychometric properties and the POS will be reported elsewhere.

## Discussion

The development and improvement of evidence-based palliative and hospice care is often limited by strong concerns that terminally ill patients and their relatives are too vulnerable to participate in end-of-life research. A recent synthesis of the current literature reviewed the evidence regarding the experiences and views of patients, caregivers, professionals and researchers with regard to participation in end-of-life care research [5]. Focusing on caregivers as study participants, the authors summarise that participating in research was not a negative experience for most of them, although a minority also experienced distress regarding the reported burden caused by interview participation. The main reason for perceived burden was the reported reminiscence of dying and death which was interpreted as an appropriate expression of grief and bereavement by the authors. In contrast, some benefits from and motivations for participation were mentioned. In the literature, the experience of distress was predominantly related to participants’ characteristics, the type of research and the way in which the study was implemented [5].

The findings from this questionnaire validation study involving informal caregivers strongly support this overall perspective. The participation rate in the presented study (76.1%) was relatively high. This may be due to few exclusion and rather broad inclusion criteria for informal caregivers, but may also be because of a very personal invitation by a trained psychologist via telephone. It is likely that the phone call was able to build on previous experiences and feelings of connectedness to members of the palliative care team. Additionally, the phone call may have offered the opportunity to directly discuss questions, to express reservations and to provide more detailed information on the study which may have led to a higher participation rate than with written study invitations. Comparable studies using the same questionnaire where no former personal contact to the potential study participants existed had considerably lower participation rates. Curtis et al. [19] sent invitation letters as the primary form of contact and then telephoned to ask if the recipient, or one of the patient’s other family members, would complete an interview in person. In this study, 36.4% of the family members of those contacted participated in the study. However, inviting family members by way of a phone call may increase social desirability for consenting to research participation and has to be considered carefully when planning end-of-life care research.

Aspects which contribute to this research challenge include the exact definition of informal caregivers, the point in time at which they are presented with and informed of a particular study, the particular manner in which recruitment should take place. The study design and its specific methods have been confirmed as being well adapted and flexible enough to enable bereaved informal caregivers to participate in end-of-life research. Although a very sensitive issue, the quality of dying and death was addressed, leading to the conclusion that the method of recruitment and the way in which the study was ultimately conducted led to a satisfactory high recruitment rate. The point in time of the study invitation being at the end of the fourth week after the patients’ death at the earliest, the method of personal interviews, and the relatives’ free choice of whether to meet at the palliative care unit or in their private home were highlighted as valuable elements by the participants. Some participants even stated that they benefited from participation and found it helpful to talk about and reflect on the issue. Comparable results can be found in current literature [4,10,20-22]. Participant feedback seems to be especially positive in research using surveys, interviews, observational methods or participatory methods. In a study by Davies et al. most family members (96%) consented to interview participation and 63% reported receiving comfort from being interviewed, while only 5% disliked participation [23]. Emanuel et al. conducted a study to assess whether interviewing terminally ill patients and their caregivers about death, dying, and bereavement is stressful or helpful. 97.6% of eligible family caregivers were interviewed and afterwards 9.9% of them reported great or some stress and 89.7% little or no stress. Almost a fifth of them reported that the interview was very helpful, and 34.3% considered it somewhat helpful and a follow-up interview two to six months later slightly less helpful than the first one [4]. In several other studies the majority of family caregivers did not experience psychosocial research as either a burden or as beneficial, and distress due to being interviewed about end-of-life issues was only reported by a minority [10,22]. In several cases, family caregivers reported that they explicitly support end-of-life care research and measures of quality management and that they were highly willing to help. This is in accordance with findings from the literature. Gysels et al. [21] found altruism as one of the main motivations for study participation.

The inclusion rate for the second phone call was as high as for the first phone call and the majority of the initially unsure candidates could be included. Therefore, the option of calling informal caregivers a second time to invite them to participate in a study has been proven to be a desirable secondary effect. Nevertheless, this option has to be conducted very carefully. If caregivers are not willing to be involved at all during the first phone call, this decision has to be respected and they should not be asked again. For participants who feel unsure of how to decide or who are overwhelmed at the time of the first phone call, it seems to be an appropriate strategy to give them more time for reflection and questions until the second call.

Reflecting on the disadvantages of the method used in the validation study here was rather time consuming and required a relatively high number of research staff. Hunt et al. [24] compared two family caregiver study groups: one so-called ‘opt in’ group where a letter of invitation was issued with a reply slip to request a copy of the questionnaire and one ‘opt out’ group where the survey questionnaire was provided with the invitation letter. These two groups were assessed for response rate and distress. The study concludes that the response rate was significantly higher in the ‘opt out’ group (40%) than in the ‘opt in’ group (26.4%). No differences were found in distress parameters, meaning that the ‘opt out’ method could be recommended as the most efficient way to recruit for surveys, even for those of a sensitive nature [24]. Nevertheless, when this is compared with our study, we see that the response rate was higher using phone calls. Therefore, it could be worth investing in more research staff.

In summary, the recruitment strategy resulted in a high participation rate. Additionally, the perceived burden caused by the research interview and the time requirements for study participation was judged acceptable by the authors. Participants’ comments hint at benefits that participants experienced from participation in this study. For the group of participants who perceived a higher burden level the framework of support available was necessary.

### Study limitations

This study provides no information on informal caregivers who did not respond, as it was highly difficult to assess demographic data when refusal was stated during the invitation phone call. This manuscript presents data from a single multi-centre validation study, which does not allow for comparison to other study designs. This limitation could affect the generalisability to other designs, populations, settings and research issues.

The authors were trained psychologists and were sensitive to communication, to notice burden and to adapt to the situation of study participation in a respectful and protective manner. However, using trained staff does not fully ensure that caregivers did not feel any social desirability or feel obliged to participate in this research project. A minor limitation is that the same researchers who conducted the study also asked the participants about their experience of the study which could have resulted in over-positive responses due to social desirability bias.

## Conclusions

In summary, evidence from the study presented here shows that ethical concerns against end-of-life research on sensitive issues with bereaved family caregivers are somewhat unjustified. The method used which has been demonstrated and successfully implemented in this population, can be recommended to other researchers and clinicians developing bereavement studies, although large efforts of personnel have to be taken into account.

Bereaved caregivers of deceased patients are willing to discuss dying and death in a structured interview with overall low to moderate levels of burden and report that the personal interview is helpful in some way. The overall performance of caregivers shows that this study design is applicable. Therefore, institutional ethical review boards should not pre-emptively restrict surveys or interviews with bereaved caregivers in case the caregivers withdraw their consent at any time during participation.

On the contrary, some family caregivers are willing to support end-of-life care research and have different motivations for participation. The reported benefits of participation should be considered as valuable perspectives on the involvement of informal caregivers in palliative care research.

## Abbreviations

QoDD: Quality of Dying and Death
PHQ: Patient Health Questionnaire, German version
POS: Palliative Care Outcome Scale
SD: Standard deviation
PCU: Palliative Care Unit

## References

[1] Aktas A, Walsh D (2011). Methodological challenges in supportive and palliative care cancer research. Semin Oncol.

[2] Rees E (2001). The ethics and practicalities of consent in palliative care research: an overview. Int J Palliat Nurs.

[3] Borgsteede SD, Deliens L, Francke AL, Stalman WA, Willems DL, van Eijk JT (2006). Defining the patient population: one of the problems for palliative care research. Palliat Med.

[4] Emanuel EJ, Fairclough DL, Wolfe P, Emanuel LL (2004). Talking with terminally ill patients and their caregivers about death, dying, and bereavement: is it stressful? Is it helpful?. Arch Intern Med.

[5] Gysels MH, Evans C, Higginson IJ (2012). Patient, caregiver, health professional and researcher views and experiences of participating in research at the end of life: a critical interpretive synthesis of the literature. BMC Med Res Methodol..

[6] Jordhoy MS, Kaasa S, Fayers P, Ovreness T, Underland G, Ahlner-Elmqvist M (1999). Challenges in palliative care research; recruitment, attrition and compliance: experience from a randomized controlled trial. Palliat Med.

[7] Gysels M, Evans CJ, Lewis P, Speck P, Benalia H, Preston NJ (2013). MORECare research methods guidance development: recommendations for ethical issues in palliative and end-of-life care research. Palliat Med.

[8] Casarett D, Ferrell B, Kirschling J, Levetown M, Merriman MP, Ramey M (2001). NHPCO task force statement on the ethics of hospice participation in research. J Palliat Med.

[9] White C, Hardy J (2010). What do palliative care patients and their relatives think about research in palliative care?-a systematic review. Support Care Cancer.

[10] Takesaka J, Crowley R, Casarett D (2004). What is the risk of distress in palliative care survey research?. J Pain Symptom Manage.

[11] Hudson P (2003). The experience of research participation for family caregivers of palliative care cancer patients. Int J Palliat Nurs.

[12] Heckel M, Bussmann S, Mai S, Stiel S, Weber M, Ostgathe C (2014). Validierung des Fragebogens ‘Qualitiyof Dying and Death’ (QoDD) für Nahestehende von Palliativpatienten im deutschen Sprachraum. Z Palliativmed.

[13] Casarett DJ, Crowley R, Hirschman KB (2003). Surveys to assess satisfaction with end-of-life care: does timing matter?. J Pain Symptom Manage.

[14] Lynn J, Teno JM, Phillips RS, Wu AW, Desbiens N, Harrold J (1997). Perceptions by family members of the dying experience of older and seriously ill patients. SUPPORT Investigators. Study to understand prognoses and preferences for outcomes and risks of treatments. Ann Intern Med..

[15] Mularski RA, Heine CE, Osborne ML, Ganzini L, Curtis JR (2005). Quality of dying in the ICU: ratings by family members. Chest.

[16] Bausewein C, Fegg M, Radbruch L, Nauck F, van Mackensen S, Borasio GD (2005). Validation and clinical application of the german version of the palliative care outcome scale. J Pain Symptom Manage.

[17] Gräfe K, Zipfel S, Herzog W, Löwe B (2004). Screening psychischer Störungen mit dem “Gesundheitsfragebogen für Patienten (PHQ-D)”. Ergebnisse der deutschen Validierungsstudie. Diagnostica..

[18] Stiel S, Matthes ME, Bertram L, Ostgathe C, Elsner F, Radbruch L (2010). [Validation of the new version of the minimal documentation system (MIDOS) for patients in palliative care : the German version of the edmonton symptom assessment scale (ESAS)]. Schmerz.

[19] Curtis JR, Patrick DL, Engelberg RA, Norris K, Asp C, Byock I (2002). A measure of the quality of dying and death. Initial validation using after-death interviews with family members. J Pain Symptom Manage..

[20] McClement SE, Woodgate RL (1998). Research with families in palliative care: conceptual and methodological challenges. Eur J Cancer Care (Engl).

[21] Gysels M, Shipman C, Higginson IJ (2008). “I will do it if it will help others”: motivations among patients taking part in qualitative studies in palliative care. J Pain Symptom Manage..

[22] Pessin H, Galietta M, Nelson CJ, Brescia R, Rosenfeld B, Breitbart W (2008). Burden and benefit of psychosocial research at the end of life. J Palliat Med.

[23] Davies EA, Hall SM, Clarke CR, Bannon MP, Hopkins AP (1998). Do research interviews cause distress or interfere in management? Experience from a study of cancer patients. J R Coll Physicians Lond.

[24] Hunt KJ, Shlomo N, Addington-Hall J (2013). Participant recruitment in sensitive surveys: a comparative trial of ‘opt in’ versus ‘opt out’ approaches. BMC Med Res Methodol..

